# Molecular mechanisms of cancer metastasis via the lymphatic versus the blood vessels

**DOI:** 10.1007/s10585-021-10120-z

**Published:** 2021-11-12

**Authors:** Stanley P. Leong, Kamila Naxerova, Laura Keller, Klaus Pantel, Marlys Witte

**Affiliations:** 1 California Pacific Medical Center and Research Institute, San Francisco, CA USA; 2 Center for Systems Biology, Department of Radiology, Massachusetts General Hospital, Harvard Medical School, Boston, MA USA; 3 Institute of Tumor Biology, University Medical Center Hamburg-Eppendorf, Hamburg, Germany; 4 Department of Surgery, Neurosurgery and Pediatrics, University of Arizona College of Medicine-Tucson, Tucson, AZ USA; 5 University of California, San Francisco, San Francisco, CA USA

**Keywords:** Cancer metastasis, Cancer metastasis through the lymphatic vessels, Cancer metastasis through the blood vessels

## Abstract

Cancer metastasis is the process by which primary cancer cells invade through the lymphatic or blood vessels to distant sites. The molecular mechanisms by which cancer cells spread either through the lymphatic versus blood vessels or both are not well established. Two major developments have helped us to understand the process more clearly. First, the development of the sentinel lymph node (SLN) concept which is well established in melanoma and breast cancer. The SLN is the first lymph node in the draining nodal basin to receive cancer cells. Patients with a negative SLN biopsy show a significantly lower incidence of distant metastasis, suggesting that the SLN may be the major gateway for cancer metastasis in these cancer types. Second, the discovery and characterization of several biomarkers including VEGF-C, LYVE-1, Podoplanin and Prox-1 have opened new vistas in the understanding of the induction of lymphangiogenesis by cancer cells. Cancer cells must complete multiple steps to invade the lymphatic system, some of which may be enabled by the evolution of new traits during cancer progression. Thus, cancer cells may spread initially through the main gateway of the SLN, from which evolving cancer clones can invade the blood vessels to distant sites. Cancer cells may also enter the blood vessels directly, bypassing the SLN to establish distant metastases. Future studies need to pinpoint the molecules that are used by cancer cells at different stages of metastasis via different routes so that specific therapies can be targeted against these molecules, with the goal of stopping or preventing cancer metastasis.

## Introduction

### Stanley P. Leong

Halsted introduced radical axillary lymph node dissection for breast cancer to control cancer spread as he believed that axillary metastasis served as a route of systemic disease [1]. His observation implied that cancer could be “cured” by radical resection of the primary cancer and regional lymph node dissection. Thus, radical regional lymph node dissection became standard for different types of cancer including breast cancer, melanoma, esophagus, gastric, colon, gynecological cancers and others. However, systemic recurrence still occurred despite adequate resection of the primary site and radical regional lymph node dissection. Based on the breast cancer model, Fisher proposed that breast cancer, by the time it was clinically apparent, was already a systemic disease [2]. Small cancer was just an early manifestation of such systemic disease, which, if it were to metastasize, would have already metastasized. Lymph node involvement was not an orderly contiguous extension, but rather a marker of distant disease. Subclincal disease was referred to as micrometastases. Under these circumstances, treatment of local or regional disease should not affect survival. Therefore, Cady asserted that the lymph node was the marker but not the governor of cancer metastasis [3]. An alternate spectrum theory by Hellman was developed to explain the clinical phenomenon of oligometastasis that cancer development was progressive. Not only was there a spectrum of malignancy, but there was an accompanying spectrum of potential curative treatments. This paradigm emphasized the importance of specific characteristics related to where in the spectrum of malignancy an individual cancer was compartmentalized. Truly localized, oligometastatic and widely metastatic cancers were likely to require different treatment strategies. Appropriate treatment consisting of surgery or radiation therapy would allow curative treatment of such oligometastases either alone or combined with systemic therapy [4].

The dilemma of assessing the regional lymph node and yet to avoid a radical lymph node dissection was resolved by the work of two sentinel lymph node (SLN) surgical pioneers, Cabanas [5] using the penile carcinoma model and Morton [6] using the melanoma model. They have established the SLN concept and the practice of SLN biopsy in the staging of regional nodal basin. Based on the anatomy and physiology of SLN, Reintgen concluded that cancer metastasis from the primary site to the SLNs is an orderly process [7]. The significant implication from the SLN principle is that when the SLN biopsy is negative, a radical lymph node dissection with increased morbidity can be spared. Patients with a negative SLN biopsy enjoy a significantly improved survival [8].

From a recent multicenter SLN Working Group study of 4059 melanoma patients with a negative SLN biopsy with a median follow-up of 31.2 months, it was found that the distant metastatic rate was 4.7% [9]. In another retrospective study with 1277 melanoma patients with a negative SLN biopsy, it was found that 126 patients developed systemic disease (9.9%) [10].

Thus, in about 5–10% of the patients with primary melanoma the initial metastatic route may bypass the SLN, likely through blood vessels to reach the distant sites. But, most of the patients with a negative SLN biopsy were disease-free. It has been shown that melanoma patients with a negative SLN biopsy have low recurrence rate of 5–10% [9, 10] as stated above. For breast cancer patients with a negative SLN biopsy, their recurrence rate is also very low, less than 5% [11]. Overall, melanoma or breast cancer patients with a negative SLN biopsy fare much better than those with a positive SLN biopsy [12, 13].

When a SLN is positive with micrometastais, is it an incubator for cancer cells to proliferate before spreading to the distant sites or is it a marker for systemic disease [10]? Perhaps, there is a spectrum of the SLN being an incubator then to a marker, when the tumor burden reaches a certain degree with associated distant metastais. The cut off level of tumor burden for this transition has not been determined. In the post-sentinel node era, we have learned that, in most cases such as melanoma and breast cancer, cancer in the early stage spreads in an orderly process from the primary site to the SLNs primarily and then beyond to the distant sites [7].

As described above based on the clinical outcome of melanoma and breast cancer patients with a negative SLN biopsy having a low rate of distant metastasis, it can be induced that the SLN may be the major gateway for metastasis to the distant sites. Nathanson et al. suggest that breast cancer cells may invade the lymphatic system at least most of the time rather than the blood vessels at the primary site. Once the breast cancer cells reach the sentinel lymph node, they may gain direct access to the systemic circulation to the distant sites by invasion into the veins in the lymph node [14].

We might ask whether the same clones of cancer cells that invade lymphatics also invade blood vessels, or are they different? It is apparent that the lymphatic system draining different body organs are different, some with more complex network than the others. The SLN drainage of melanoma and breast cancer follows more reliable routes than the internal organs such as the lungs, stomach, pancreas and other organs, which show complicated lymphatic drainage patterns. Certainly, melanoma and breast cancer allow us to appreciate the biology of early cancer arriving in the SLNs, then to the non-sentinel lymph node compartment and beyond to the distant sites. Based on the Rotterdam criteria, developed by van Akkooi et al., they showed that melanoma patients with increasing SLN tumor burden had a worse survival and higher incidence of metastasis in the non-SLN basin in the completion lymph node dissection. Patients with micrometastases (≤ 0.1 mm) had an excellent outcome, similar to SLN-negative patients. However, patients with larger metastases (≥ 1 mm) had a poor prognosis, similar to patients with palpable nodal disease [15, 16]. Similarly, in breast cancer patients, if the sentinel lymph node is found to harbor isolated tumor cells (ITC; < or = 0.2 mm), their clinical outcome is the same as those patients with a negative SLN biopsy [17]. Therefore, the spectrum theory seems to be most compatible with the clinical outcome of melanoma and breast cancer patients undergoing SLN staging procedure with a negative SLN biopsy or with a minimal tumor burden in the SLN.

**Fig. 1:**
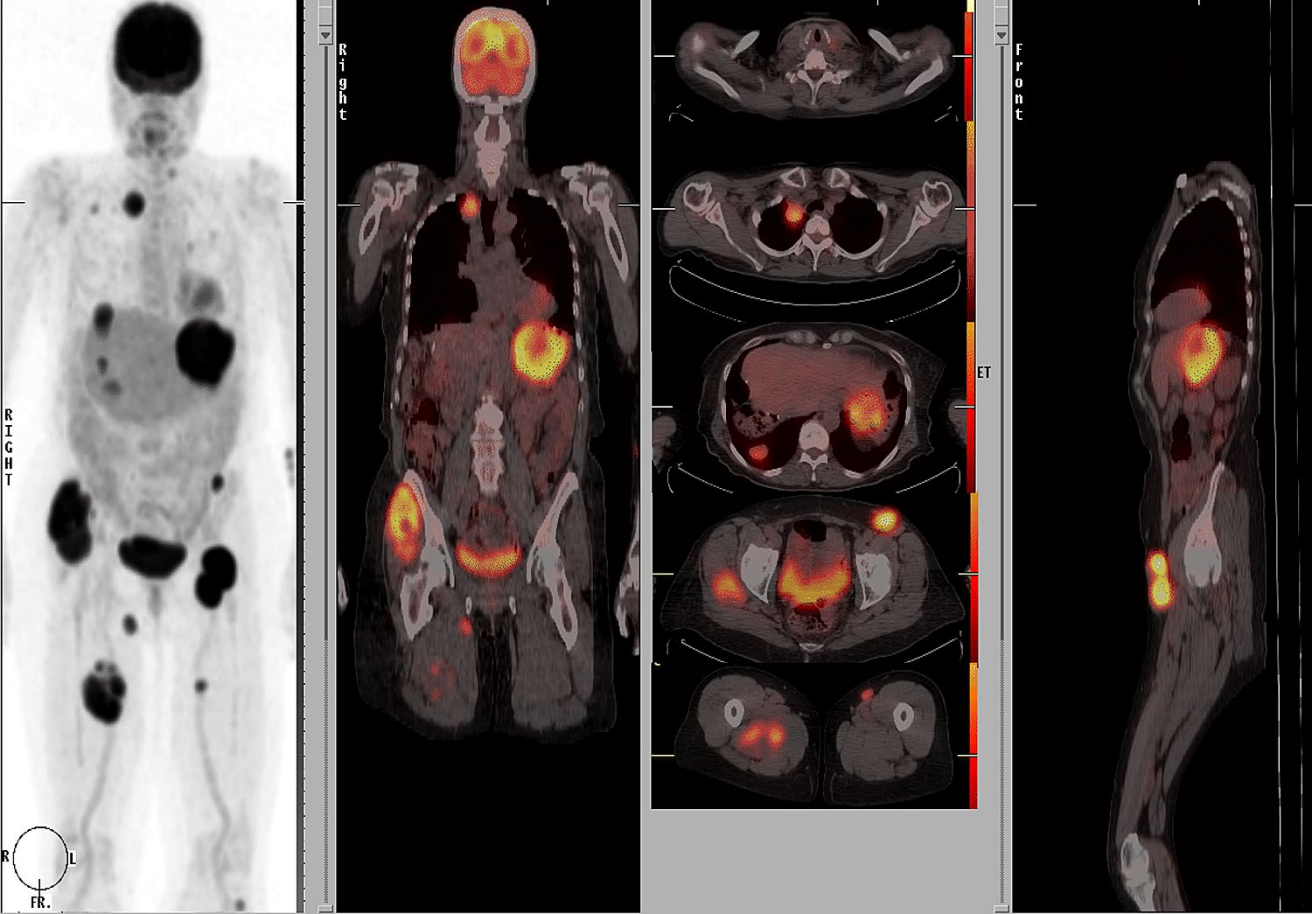
61 year old Caucasian woman with disseminated melanoma by PET/CT scan, previously published as a book cover, From Local Invasion to Metastatic Cancer: Involvement of Distant Sites Through the Lymphovascular System, Ed. Stanley P. L. Leong, Humana Press, 2009, New York, NY, USA

**Fig. 2:**
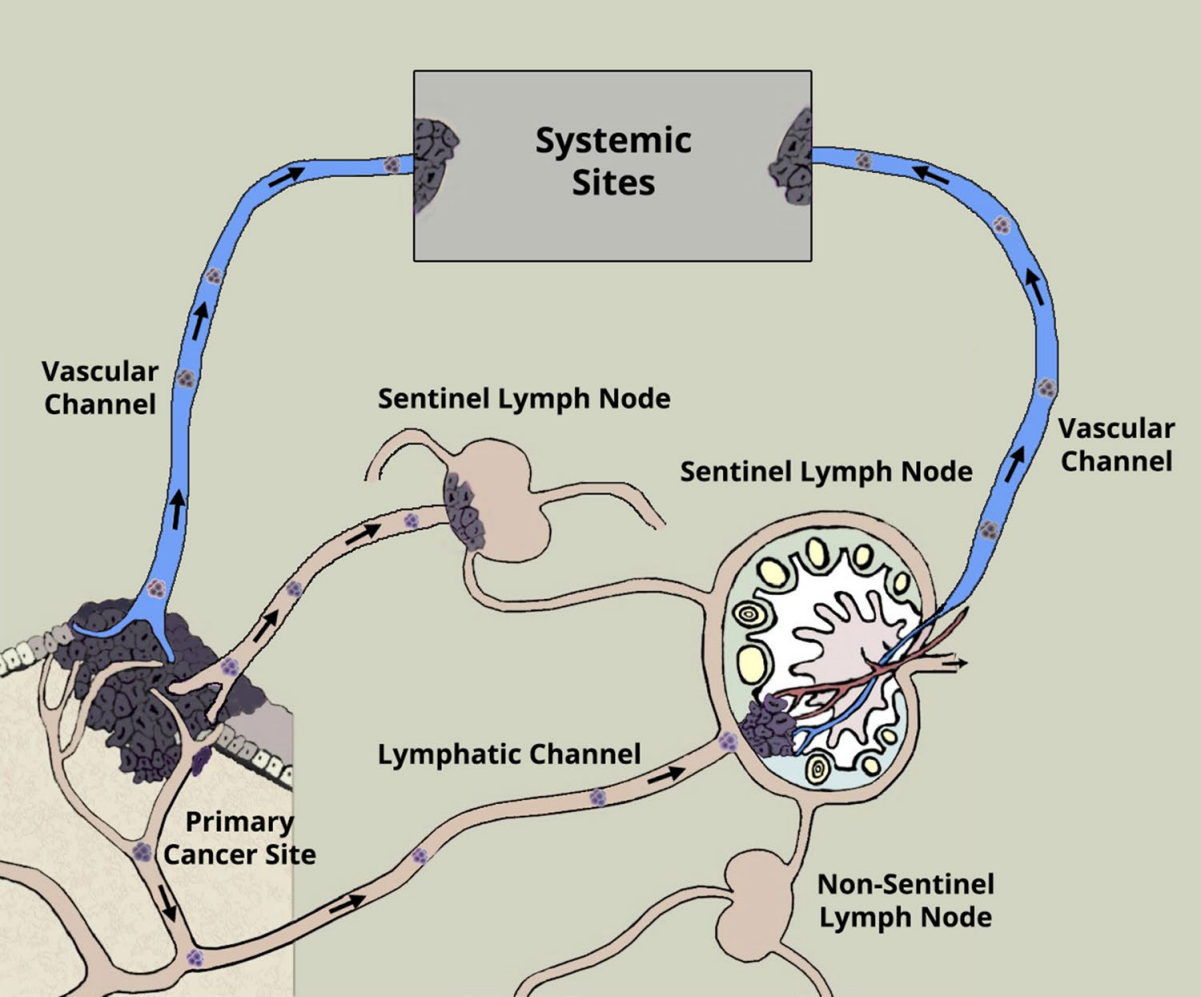
Dichotomy of routes of cancer metastasis: one through the lymphatic vessels to the sentinel lymph nodes as the primary gateway and the other through the blood vessels directly to the distant sites. Permission has been obtained to reproduce this figure from Springer Nature from the cover image for the Special Issue of Clinical and Experimental Metastasis, Springer Nature, Volume 35, Number 5–6, 2018

**Fig. 3:**
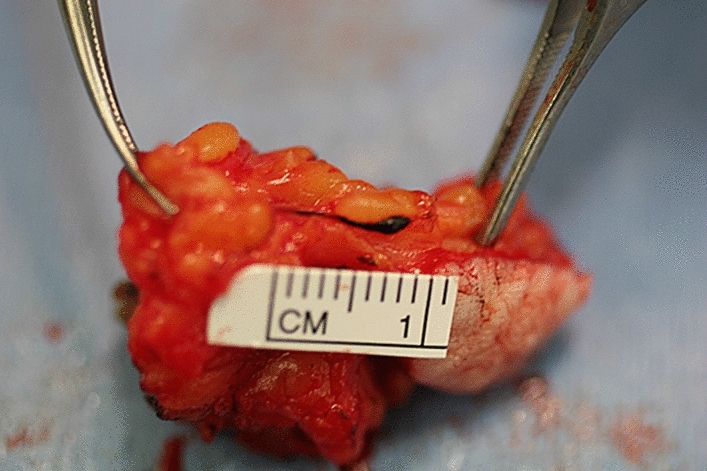
Metastatic melanoma encased in the lymphatic channel within the subcutaneous tissue

**Fig. 4:**
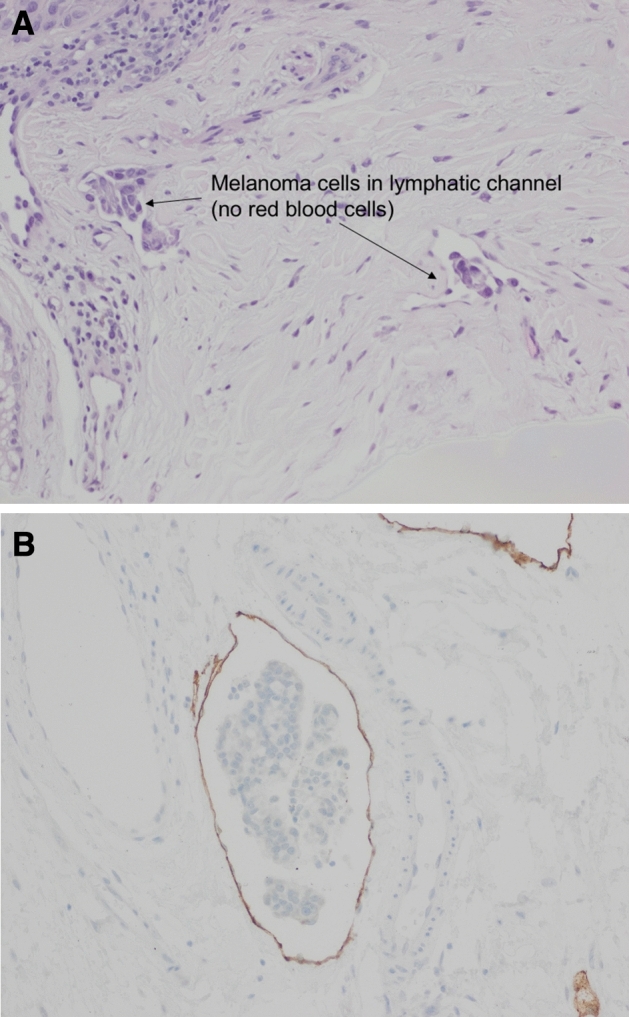
**A** H&E staining of melanoma cells involving the lymphatic space (200X) as suggested by the lack of red blood cells in the endothelial space. **B** Breast carcinoma involving lymphatic space (200x). D2-40 (podoplanin) lymphatic endothelial marker stain supports lymphatic invasion of breast carcinoma

In this review article, we attempt to address the molecular mechanisms of cancer spread from the primary site to the distant sites (Fig. 1) by the lymphatic or blood vessels. Is the SLN the gateway for systemic metastasis for most patients? What is the spectrum of tumor burden in the SLN to initiate systemic metastasis? Fig. 2 illustrates the dichotomy of routes of cancer metastasis through the lymphatic versus blood vessels. Occasionally, the cancer cells may be caught in the lymphatic vessel as in in-transit metastatic melanoma as shown in Fig. 3. Figure 4A and B show cancer cells localized within the lymphatic vessels under microscopic examination.

This review article captures the Session on Molecular Mechanisms of Cancer Spread via the Lymphatic versus the Blood Vascular Channels being presented at the 8th International Cancer Metastasis Congress in San Francisco, CA, USA from October 25–27, 2019 (www.cancermetastasis.org). Stanley Leong describes mechanisms of metastasis being promoted by lymphatic endothelium based on the presentation of Michael Detmar and the “yin and yang” concept of tumor-associated lymphatics in metastasis and immunotherapy from that of Melody Swartz. Kamila Naxerova utilizes genomic studies to describe the phylogenetic origins of lymphatic and distant metastasis in colorectal cancer. Laura Keller and Klaus Pantel address the metastatic process in relationship to cancer micrometastasis and dormancy. Marlys Witte summarizes the findings of this review and gives us the perspectives for future studies.

## Mechanism of promotion of distant metastasis by lymphatic endothelium

### Stanley P. Leong

Although the clinical significance of SLNs in melanoma and breast cancer has been well established [10, 11, 18], the molecular mechanism as how the cancer cells migrate to the SLNs has not been well characterized. With the discovery of the vascular endothelial growth factor (VEGF) family members and their receptors in 1983 [19] and the subsequent cloning of the gene in 1989 [20, 21], the molecular mechanism of cancer spread has become better defined using these molecules. VEGFs represent a family of secreted polypeptides. They contain a highly conserved cystine receptor-binding structure similar to that of the platelet-derived growth factors. The original member of the family, VEGF-A, is present up the phylogenetic tree from primitive fish to highly evolved mammals. VEGF-A plays a critical role in blood vessel formation for embryonic vasculogenesis and angiogenesis. It is also responsible for neovascularization in cancer and other diseases [22]. VEGF-A is the prototypical member of a family of related growth factors that includes placental growth factor (PLGF), VEGF-B, VEGF C, and VEGF-D, and the viral VEGF-Es [23–25]. The biological functions of the VEGFs are mediated by a family of protein tyrosine kinase receptors (VEGFRs) [26–28]. The primary biological role of VEGF-C, discovered in 1996 as a ligand for the orphan receptor VEGFR-3, is to initiate the growth and proliferation of lymphatic vessels, a process termed lymphangiogenesis. It interacts with lymphatic endothelial cells (LECs) primarily via its receptor VEGFR-3 promoting survival, growth and migration of lymphatic vessels [29].

Lymphatic vessel endothelial hyaluronan receptor 1 (LYVE-1) is a type I integral membrane glycoprotein, discovered in 1999 and encoded by the lymphatic vessel endothelial hyaluronan receptor 1 (LYVE-1) gene [30]. It is a Link domain-containing hyaladherin, a protein that binds to hyaluronic acid (HA), homologous to CD44, the main HA receptor. Like CD44, the LYVE-1 molecule binds both soluble and immobilized HA. However, unlike CD44, the LYVE-1 molecule binds with HA on the luminal face of the lymphatic vessel wall. Further, it is usually absent from blood vessels. Thus, LYVE-1 is a powerful specific marker for lymphatic vessels [30]. LYVE-1 has also been found in other tissues [31–33].

Podoplanin is a small, extensively O-glycosylated, type I transmembrane glycoprotein first discovered in rats in 1997 [34]. It is expressed by LECs but not by blood vascular endothelial cells and is therefore a widely used lymphatic-specific marker. Prox-1, a homeobox gene, was initially cloned by homology to the *Drosophila melanogaster* gene, prospero and is associated with the development of the lymphatic system [35, 36]. It has been found that Prox-1 is present in a variety of tissues, including the lens, heart, liver, pancreas and central nervous system [35]. Prox-1 expression may result in inducing the formation of lymphatic vessels from blood vascular endothelium [37, 38]. Both Prox-1 and podoplanin are mucin-type transmembrane proteins being expressed in LECs. It has been hypothesized that podoplanin functions downstream of Prox-1. Further, it appears that Podoplanin expression is regulated by Prox-1 in the LECs at the transcriptional level [39]. At any rate, both podoplanin and PROX-1 are excellent markers for identification of lymphatic vessels.nter.

To date, there are several excellent lymphatic vessels biomarkers including VEGF-R3, LYVE-1, podoplanin and Prox-1, as described above, being used to identify lymphatic vessels or LECs and to study the process of lymphangiogenesis [40]. These markers have been employed to detect lymphangiogenesis and lymphatic vessel invasion by cancer [41–43].

Detmar et al. have launched a series of experiments to show that lymphagniogensis is present during cancer transport and invasion in the murine models and human. With the availability of the lymphatic markers, Skobe and Detmar et al. have shown that VEGF-C induces tumor lymphangiogenesis, thereby, promoting breast cancer metastasis [44].

Using LYVE-1, Skobe et al. showed the development of intratumoral lymphangiogenesis within human breast cancers following orthotopic transplantation of breast cancer in nude mice. They found that VEGF-C overexpression in breast cancer cells was associated with increased intratumoral lymphangiogenesis, which was highly correlated with the extent of lymph node and lung metastases. Thus, they concluded that VEGF-C could be a molecular link between cancer lymphangiogenesis and metastasis.

In cutaneous melanoma, dual staining with LYVE-1 and the panvascular marker CD31 was used by Dadras and Detmar [45]. Eighteen melanoma samples were obtained from clinically and histologically closely matched cases of primary melanomas with early nodal metastasis and 19 samples from nonmetastatic melanomas were examined. They found that the incidence of intratumoral lymphatics was significantly higher in metastatic melanomas. Increased intratumoral lymphatics were associated with poor disease-free survival. A relative lymphatic vessel area of > 1.5% was significantly correlated with poor disease-free and overall survival. On the contrary, they found that there were no differences in the density of tumor-associated blood vessels. They proposed that intratumoral lymphangiogenesis was a novel prognostic marker for the risk of lymph node metastasis in melanoma [45]. Numerous studies have shown that increased lymphagniogenesis associated with increased intratumoral lymphatics and lymphatic invasion is correlated significantly with reduced overall survival in melanoma, breast, colorectal and lung cancer. The generation of new lymphatic vessels through lymphangiogenesis and the remodeling of existing lymphatics are thought to be important steps in cancer metastasis [46].

**Fig. 5:**
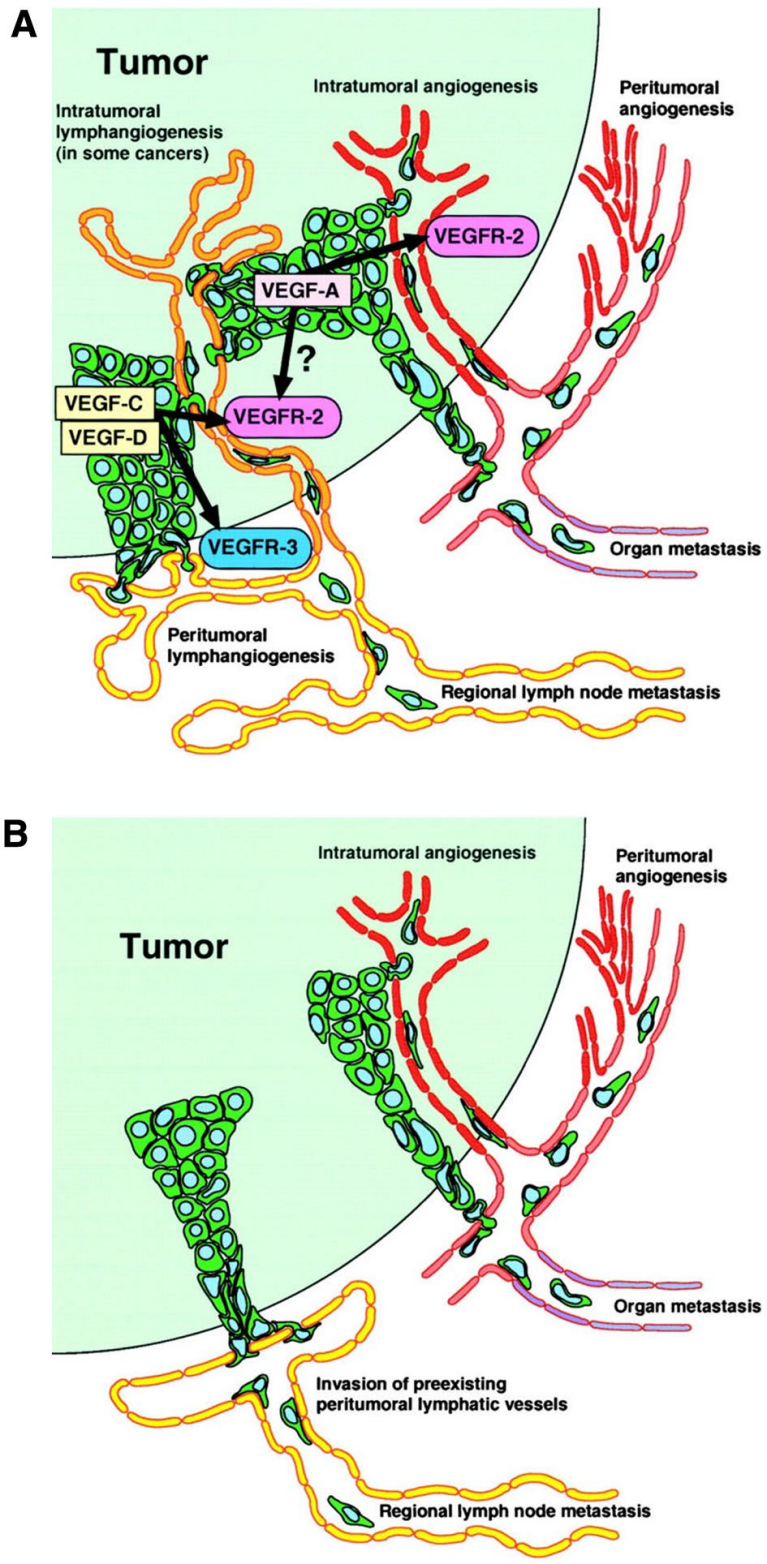
**A** Active lymphangiogenesis model of tumor metastasis. **B** Traditional model of tumor metastasis via lymphatic and blood vessels. [47]. Permission has been obtained from J Exp Med to reproduce these figures

Based on the association of lymphagniogenesis and cancer metastasis in animal models and human studies, Detmar and Hirakawa have proposed the lymphangiogenesis model of cancer metastasis (Fig. 5A) with VEGF-C and VEGFR-3 axis playing an important role in directing and allowing the cancer cells to spread through the lymphatic vessels [47].

It has been shown that cancer-induced lymphatic vessels by means of lymphangiogenesis may become dilated [48]. A cluster of cancer cells may get caught at the valve in the lymphatic vessel wall [49] with eventual growth on the inner wall of the lymphatic vessel as a colony to invade into the surrounding soft tissue as in-transit metastasis, as in melanoma (Fig. 2).

Thus, cancer cells may “hitch hike” the lymphatic system to create passages for cancer metastasis by expressing growth factors, which alter the normal pattern of lymphatic vessel growth during lymphangiogenesis (Fig. 5A) and angiogenesis (Fig. 5B). Recent identification and characterizations of lymphatic biomarkers have ushered new directions of research in the anatomy and physiology of lymphatic vessels in general and in cancer metastasis particularly. These molecular targets may potentially be used for therapeutic benefits to control or stop cancer metastasis [50]

Fujimoto et al. used single-cell RNA sequencing to define the comprehensive transcriptome of LECs in murine skin-draining lymph nodes [51]. New markers and functions of distinct LEC subpopulations were identified. LECs residing in the subcapsular sinus have been found to have a unique function in scavenging of modified low-density lipoprotein (LDL). A specific cortical LEC subtype was identified being associated with rapid lymphocyte egress from lymph nodes. These findings of LEC diversity and functions are important for future studies regarding the regulation of immune responses by lymph node LECs [51]. Recently, it has been demonstrated that LECs may respond and affect the immune response [52].

Under normal physiological conditions, LECs in the lymph node have been found to maintain peripheral tolerance by directly inhibiting T-cells against autologous antigens. It has been suggested that tumor-associated LECs may reduce T-cell responses against autologous tumors. Using two distinct experimental tumor models, Dieterich et al. have found that tumor-associated LECs express the T-cell inhibitory molecule programmed death-ligand 1 (PDL-1), like LECs in the lymph node. On the other hand, PDL-1 is downregulated in tumor-associated blood vessels. It was speculated that the observed lymphatic upregulation of PDL-1 was probably due to gamma interferon-g released by stromal cells in the tumor microenvironment. Further, the authors have found that increased T-cell stimulation by antigen-presenting LECs in vitro emerges when PDL-1 is blocked. Thus, the authors assert that tumor-associated lymphatic vessels play several important roles during tumor progression, such as initiation of metastasis and modulation of immune responses against autologous tumor by sending antigen from the tumor microenvironment to draining lymph nodes. They suggest that peripheral tumor-associated lymphatic LECs induce T-cell inhibition, by a mechanism akin to maintenance of peripheral tolerance by resident LECs in lymph nodes. Since PDL-1 has been implicated in tumor tolerance, it may be directed as a therapeutic biomarker using checkpoint inhibitors such as anti-programmed cell death protein 1 or anti-PDL-1 [53].

Using a transgenic mouse model with a standard chemically-induced skin carcinogenesis regimen, Hirakawa et al. have found that overexpressed VEGF-A in the skin not only strongly promotes multistep skin carcinogenesis, but also promotes active proliferation of VEGF receptor-2–expressing tumor-associated lymphatic vessels, and furthermore, tumor metastasis to the sentinel and distant lymph nodes. This newly identified mechanism of tumor cells expressing VEGF-A–expressing tumor cells being present in metastatic lymph nodes is intriguing. In addition, VEGF-A–overexpressing primary tumors induced SLN lymphangiogenesis even prior to metastasizing to the SLN. The authors suggest that lymphangiogenic factors may be delivered to the SLN to prepare for the future arrival of the metastatic cancer cells [54].

Further work on the metastatic niche in draining lymph nodes was carried out in 2 mouse models with the 4T1 breast cancer and B16F10 melanoma by Commerford et al. [55]. They have found as early as 4 days after tumor injection, LEC sprouting and proliferation in tumor-draining lymph nodes representing lymphatic expansion. RNA sequencing in both tumor models showed an altered transcriptional profile of LECs from tumor-draining lymph nodes as compared to naive lymph nodes. Integrin αIIb was found to be upregulated in LECs of tumor-draining lymph nodes. In vitro, αIIb was shown to mediate LEC adhesion to fibrinogen. Since LEC-associated fibrinogen was also identified in draining lymph nodes in vivo, the authors suggested that integrin αIIb could play an important role in lymphatic remodeling. The authors noted specific LECs responses in the tumor-draining lymph nodes to tumor at the primary injection site with possible formation of metastatic niche prior to the arrival of cancer cells [55].

Furthermore, using mouse metastasis models, Ma et al. found that lymphangiogenesis occurred in distant lung metastases and that some metastatic tumor cells were located in the lymphatic vessels and draining lymph nodes of the distant metastatic sites [56]. In melanoma patients with metastatic melanoma, a higher lymphatic density was noted within and around the pulmonary metastases and lymphatic invasion were correlated with poor outcomes. Using a transgenic mouse model with inducible expression of VEGF-C in the lung, the authors found greater growth of pulmonary metastases associated with increased dissemination to other organs. They have identified the role of lymphangiogenesis in metastatic sites with further metastasis to other sites, thus, proposing the strategy of adjuvant therapy for metastatic cancer if it can be resected.

**Fig. 6:**
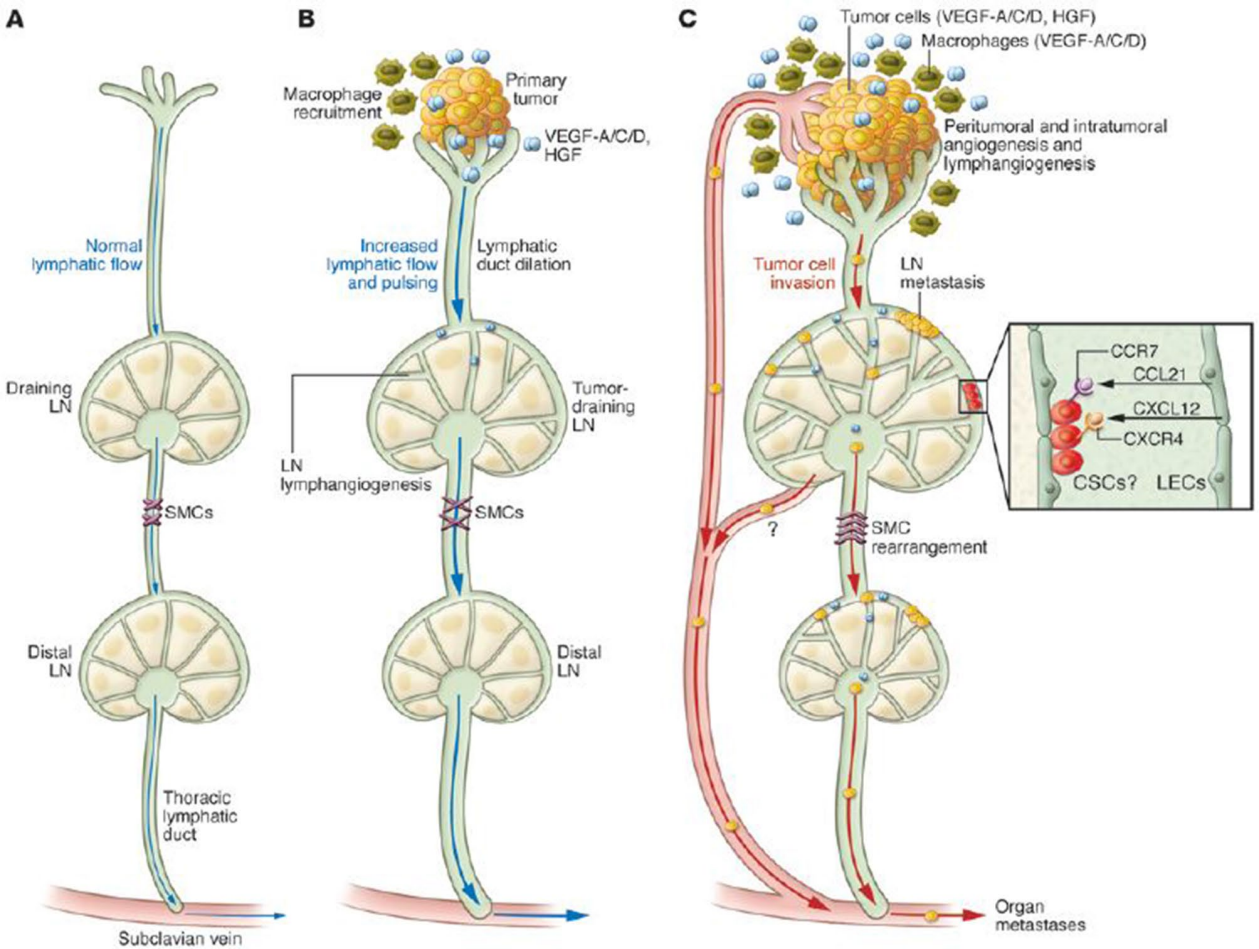
An important contribution of tumor and LN lymphangiogenesis to cancer metastasis. **A** Normal lymphatic tissue drainage through lymphatic capillaries, collecting lymphatics, and LNs. **B** lymphangiogenic factors produced by premetastatic tumors, including VEGF-C, VEGF-D, VEGF-A, and HGF, are taken up by peritumoral lymphatic capillaries and are transported via the collecting lymphatics toward the tumor-draining SLN, where they act directly on preexisting lymphatic vessels to induce LN lymphangiogenesis. Tumor-draining lymphatic vessels display an enlarged size and increased lymph flow and pulsing. (C) Once metastatic tumor cells have spread to their draining LNs, they serve as a major source of lymphangiogenic factors. These promote the remodeling and SMC rearrangement of distant (post-SLN) lymphatic vessels and lymphangiogenesis in distant LNs and promote secondary metastasis, including organ metastasis, via the thoracic duct, which connects to the venous circulation via the subclavian vein. CSC, cancer stem cell. The chemokines CCL21 and CXCL12, released by activated lymphatic endothelial cells (LECs) within SLNs, might provide a niche for cancer cells with stem cell–like properties that express the receptors CCR7 and CXCR4 [57]. Permission has been obtained to reproduce legend and figure for Fig. 6 from Journal of Clinical Investigation [57]

Karaman and Detmar assert that cancer lymphangiogenesis is induced by VEGF-C in the primary tumor microenvironment and in draining lymph nodes [57]. Lymphatic vessels do not merely serve as passive channels for tumor spread, but that they may play an active role in promoting tumor cell recruitment to lymph nodes, cancer stem cell survival and immune modulation. These important findings may open new opportunity to target these processes associated with specific biomarkers to be targeted for potential therapeutic benefits. Figure 6 explains in more detail mechanisms of tumor-associated lymphangiogenesis and cancer metastasis through the lymphatic system.

From Dr. Detmar’s presentation, it can be summarized that LECs promote cancer metastasis by several mechanisms. First, cancer-induced lymphangiogenesis results in increased lymphatic vessels with LECs being associated with enhanced lymphatic drainage to lymph nodes. Second, the so called lymphangiocrine factors may provide a metastatic lymph node niche. Third, the LECs may inhibit anti-tumor immune responses via the PDL-1 checkpoint inhibition. Fourth, the LECs may enhance distant organ metastasis resulting in further metastatic spread. It appears that lymphangiogenesis is a prognostic biomarker with an increased cancer spread and decreased survival. Can it be used as a therapeutic target?

## The yin and yang of Tumor-associated lymphatics in metastasis and immunotherapy

### Stanley P. Leong

This section has been extracted from the presentation by Melody Swartz on the yin and yang of tumor-associated lymphatics in metastasis and immunotherapy [58]. Decreased cancer survival is associated with increased lymphangiogenesis as noted in Detmar’s presentation [57, 59]. Several preclinical and clinical studies have demonstrated a positive correlation between the incidence of lymph node metastasis and secretion of the lymphatic growth factor VEGF-C by tumor cells as noted in the previous section, suggesting that lymphangiogenesis facilitates cancer metastasis through the lymphatics. However, recent studies have shown that VEGFR-3 expression on tumor cells with autocrine signaling by VEGF-3 is associated with increased metastatic potential. Also, recent evidence has shown that lymphatic-homing chemokine receptors, particularly C–C chemokine receptor 7 (CCR7) may play a role in lymph node metastasis. Issa et al. have found that VEGF-C acts to increase lymphatic secretion of CCL21, another lymphoid homing chemokine, which interacts with CCR7 to initiate cancer invasion toward lymphatics. Further, VEGF-C acts through autocrine signaling to enhance tumor invasiveness by increasing the proteolytic activity and motility of tumor cells in a three-dimensional matrix. In addition, VEGF-C induces lymphatic CCL21 up-regulation in vivo when VEGF-C protein was injected intradermally in the mouse. Thus, the authors emphasize the prometastatic functions of CCR7 and VEGF-C in tumors and indicate that their data show that VEGF-C promotes tumor invasion toward lymphatics by both autocrine and CCR7-dependent paracrine signaling mechanisms in addition to lymphangiogenesis induced by VEGF-C [60]. The role of CCR7 in lymph node metastasis of cancer has been further explored. Using the 3D matrix model, Shields et al. have shown that CCR7 creates a transcellular gradient of increased interstitial flow to enhance tumor cell migration into the lymphatic vessels, this mechanism is similar to that of dentritic cell trafficking [61].

Swartz et al. have found in a murine model that while lymphangiogenesis is associated with poor prognosis, it is also correlated with better response to checkpoint immunotherapy. This conundrum is termed “yin and yang” of tumor-associated lymphatics in metastasis and immunotherapy in her presentation. As noted in a previous section, VEGF-C-induced lymphangiogenesis in the tumor site correlates with metastasis and poor prognosis, VEGF-C also promotes tumor immunosuppression, suggesting that inhibiting lymphangiogenesis may reverse immunosuppression and may be clinically beneficial when combined with immunotherapy. Using a mouse melanoma model with VEGFR-3 blocking antibodies by Fankhauser and Swartz et al., the unexpected finding was that VEGF-C signaling did not suppress but enhance the response to antibody immunotherapy. This enhancement was found to be due to mediation by VEGF-C induced CCL21 associated with tumor infiltration of naïve T cells before immunotherapy as CCR7 blockade reversed the potentiating effects of VEGF-C. In human metastatic melanoma, it was found that VEGF-C gene expression was strongly associated with CCL21 and T cell inflammation. Further, serum VEGF-C concentrations were correlated with both T cell activation and expansion after peptide vaccination and clinical response to checkpoint inhibition immunotherapy. The authors suggest that VEGF-C enhances immunotherapy by attracting naïve T cells being locally activated upon immunotherapy induced tumor cell killing, and that serum VEGF-C may serve as a predictive biomarker for immunotherapy response [62].

Lymphatic drainage causes interstitial fluid flow throughout the extracellular matrix within the tissue microenvironment. Because of excessive interstitial fluid pressure in tumors, interstitial flow and lymphatic drainage are increased in the tumor margin [63, 64]. The increased interstitial flow may direct the cancer cells to migrate in the flow direction by chemotaxis from autologously secreted chemokines [61]. Interstitial flow can also affect stromal cells, causing cell and matrix alignment [65, 66], increasing fibroblast motility via matrix metalloproteinase-1 [67] and inducing myofibroblast differentiation via transforming growth factor (TGF)-beta 1 [65].

**Fig. 7:**
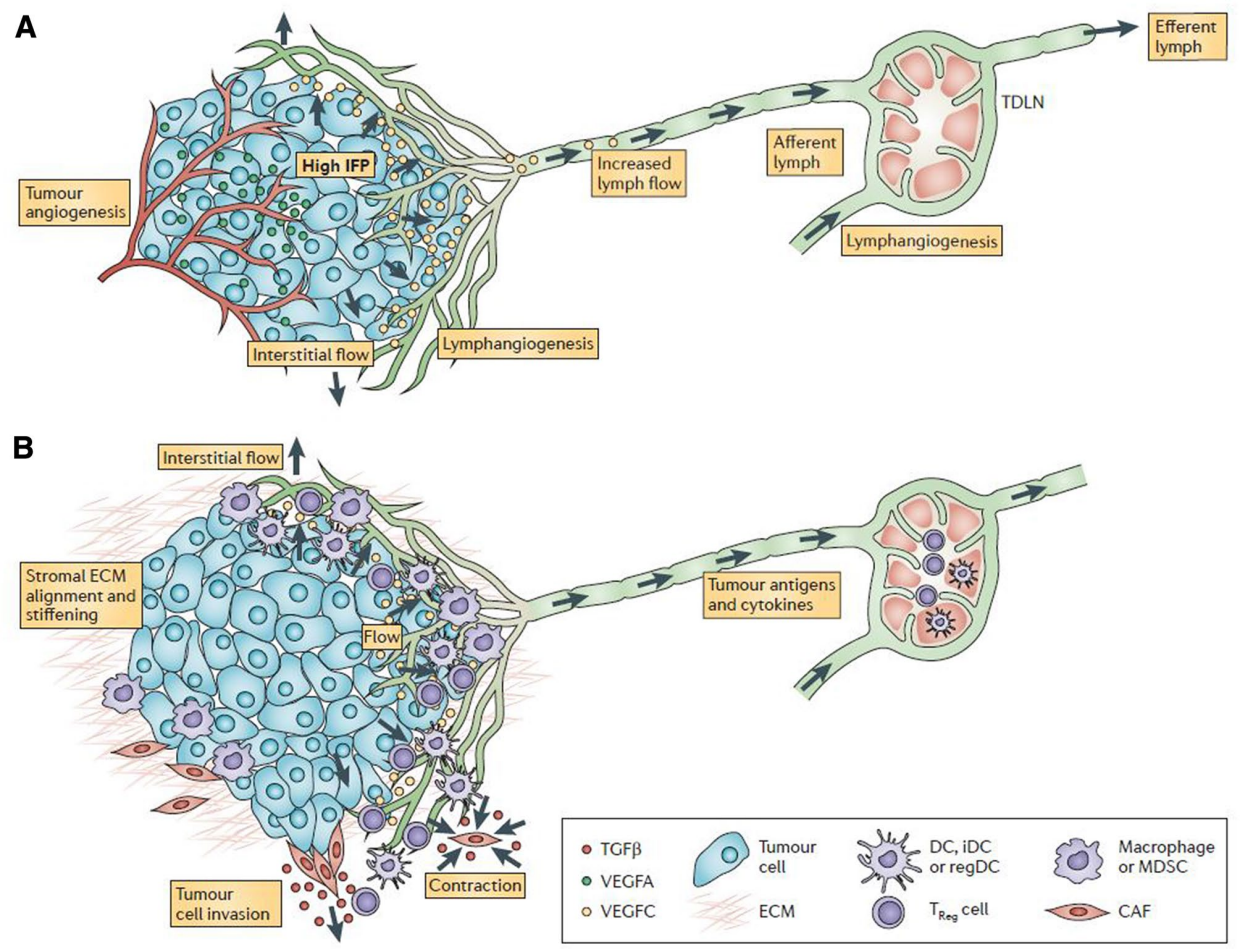
Lymphangiogenesis, mechanobiology and immunity in the tumour microenvironment contain many interdependent features. **a** | Fluid pathways in the tumour microenvironment are shown. Angiogenic, immature tumour vessels (shown in red) are hyperpermeable, driving heightened interstitial fluid pressure (IFP) in the tumour. This creates steep pressure gradients at the tumour margin that drive interstitial flow through the stroma and into peritumoral lymphatic vessels (green), which are often expanded and hyperplastic. These lymphatic vessels carry tumour interstitial fluid to the sentinel or tumour-draining lymph node (TDLN), where lymphangiogenesis is also seen. **b** | The heightened interstitial and lymphatic flows (green) in the tumour microenvironment coincide with biomechanical (red) and immunological (purple) changes in the tumour stroma. Biomechanical changes include stiffening and alignment of the extracellular matrix (ECM) of the tumour stroma, owing to cancer-associated fibroblast (CAF) contraction and remodelling that is dependent on transforming growth factor-β (TGFβ). Matrix stiffening promotes tumour cell invasion through CAF-led collective migration and activation of mechanically sensitive stromal components. Interestingly, stromal features of the tumour can mimic those of the TDLN. Immunological changes include biasing of the tumour-infiltrating lymphocyte populations by local factors. Tumour-promoting cytokines promote alternatively activated (M2) macrophages and myeloid-derived suppressor cells (MDSCs), hinder the maturation of immature dendritic cells (iDCs) and stimulate regulatory DCs (regDCs), which in turn promote regulatory T (T_Reg_) cells and suppress local cytotoxic T cell activity. Similar cell populations are biased in the TDLN. VEGF, vascular endothelial growth factor.  Permission has been obtained to reproduce legend and figure for Fig. 7 from Springer Nature: Nature Reviews Cancer, Lymphatic and interstitial flow in the tumour microenvironment: Linking mechanobiology with immunity (Swartz et al. 2012). [70]

TGF-beta 1 is produced in the cancer microenvironment by stromal cells including fibroblast and its activation transforms fibroblast to myofibroblast transition (cancer-associated fibroblast) [68] which is associated with fibroblast remodeling. Using a 3-dimensional invasion assay with cancer cell lines being cocultured with dermal fibroblasts in a collagen matrix, Shieh et al. showed tumor cell invasion was enhanced by fibroblasts in the presence of interstitial flow. This represents a novel mechanism in which increased interstitial flow in the tumor microenvironment causes fibroblast remodeling and migration through increased TGF-beta 1 activation and collagen degradation, thus, facilitating tumor invasion into the lymphatic vessels [69]. A detailed review of the cancer microenvironment linking lymphangiogenesis, interstitial flow, mechanobiology and immunity is elegantly presented by Swartz and Lund [70]. Thus, while lymhangiogenesis is associated with cancer invasion and poor prognosis, it also opens a window of therapeutic benefit with checkpoint inhibition. Figures 7A and 7B summarize the linkage between mechanobiology, lymphangiogenesis and anti-tumor immunity.

Although VEGF-C induces lymphangiogensis, enhances tumor transport to the draining lymph node and antigen exposure to the adaptive immune system, its role in tumor immunity is not clear. In this murine model of B16 F10 melanoma expressing a foreign antigen (OVA), Lund et al. has shown that VEGF-C promotes immune tolerance in murine melanoma. When mice had developed antitumor immunity against F10 with a foreign antigen, OVA, the authors found that VEGF-C conferred immune tolerance to F10 OVA immunity by deleting OVA-specific CD8 + T cells in the tumor site. When naive OVA-specific CD8 + T cells were transferred into tumor-bearing mice, they became dysfunctionally activated and apoptotic. LECS in draining lymph nodes were noted to have cross-presented OVA. In vitro, naive LECs were found to scavenge and cross-present OVA. Such cross-presenting LECs promoted the proliferation and apoptosis of OVA-specific CD8 + T cells ex vivo. The authors concluded that LECs associated with lymphangiogenesis in a tumor or the draining lymph node promoted immune tolerance by elimination of antitumor CD8 + cells. Thus, LECs in the local tumor microenvironment may be a target for immunomodulation [71].

Cancer-derived exosomes are extracellular vesicles including proteins, DNA, mRNA, microRNA, long noncoding RNA, circular RNA, etc., being associated with carcinogenesis [72]. Melanoma-derived exosomes were studied in primary tumor and metastases in mouse and human subjects. It has been found that melanoma exosomes educate bone marrow progenitor cells to become a pro-metastatic phenotype through the mesenchymal epithelial transition receptor [73]. Further, integrins from cancer exosomes have been found to determine organotropic metastasis [74]. Maillat et al. have found in a mouse model, upon intratumoral injection of fluorescent-labelled exosomes from B16F10 tumor, a significant uptake of exosomes was noted in the LECs within the tumor microenvironment and at higher levels than blood endothelial cells, with subsequent delivery of the exosomes to draining lymph nodes and eventually to the blood. In transgenic mice lacking dermal lymphatics, intratumoral injection of exosomes showed no exosomes in the lymph nodes and present in far lower amounts in the blood compared to those injected into wild type mice. Thus, the authors concluded that lymphatic vessels regulated exosome trafficking from the primary tumor site [75].

Broggi et al. have found significantly elevated tumor-derived factors such as extracellular vesicles containing melanoma-associated proteins and miRNAs, with unique protein signatures reflecting early versus advanced metastatic spread in lymphatic exudate in the postoperative wound drainage following lymphadenectomy as compared to the plasma of these postoperative patients [76]. Furthermore, lymphatic exudate was enriched in memory T cells, including tumor-reactive CD137 + and stem cell–like types. The authors suggest that lymphatic exudate provides a rich source of tumor-derived factors for enabling the discovery of novel biomarkers that may reflect disease stage and therapeutic response.

Since tumor cells and antigens preferentially are directed to the draining lymph nodes, Swartz et al. wanted to find out if tumor vaccine directed to antigen-primed tumor draining lymph nodes could be more or less effective with respect to stimulate anti-tumor immunity, despite the fact that the tumor draining lymph nodes are deemed to be immune-suppressed. Using two different murine cancer models, E.G7-OVA lymphoma (expressing the nonendogenous TAA ovalbumin) and B16-F10 melanoma, lymph node-targeting nanoparticle-conjugate vaccines were delivered to the tumor draining versus non-tumor draining lymph nodes. Despite the immune-suppressed state of the former, vaccination targeting against the tumor draining lymph nodes induced much stronger cytotoxic CD8þ T-cell responses, both locally and systemically than against the latter, resulting in enhanced tumor regression and host survival. Improved tumor regression in the mice with vaccine targeted against the tumor draining lymph nodes correlated with a change in the tumor-infiltrating leukocyte repertoire consisting of a less suppressive and more immunogenic balance. Based on this unexpected finding, the authors suggested that antigen-primed but immune-suppressed tumor draining lymph nodes could be reprogrammed with a targeted vaccine yielding antitumor immunity [77].

It has also been found that radiation to the tumor may induce immune modulation of cancer immunotherapy. As radiation induces tumor destruction, tumor antigens may be released from dying tumor cells. Moreover, radiation has been demonstrated to induce immunogenic modulation in various tumor types by altering the microenvironment of surviving cells to render them more susceptible to T cell anti-tumor activity. In addition, radiotherapy spares the patients of systemic immunosuppression. Thus, these features make radiotherapy an important adjunct to immunotherapy with the goal to achieve systemic antitumor immunity [78].

In summary, Melody Swartz has substantiated the findings from Michael Detmar’s presentation that lymphangiogenesis in the tumor microenvironment or draining lymph nodes is associated with cancer metastasis and poor survival. Further, she has shown the “yin and yang” concept of lymphagiongenesis, while it is associated with poor survival and tumor tolerance by the host, it creates an opportunity for immune modulation through checkpoint inhibition with potential therapeutic benefit. In addition, she has united lymphangiogenesis, mechanobiology of the interstitial flow within the tumor microenvironment, tumor tolerance and principles of radiotherapy into a multifactorial approach to evaluate lymphangiogenesis and develop new strategies for cancer immunotherapy.

Cancer angiogenesis has been well established as mechanism for cancer to take up nutrients to grow as well as a viable explanation for cancer cells to spread via the newly formed blood vessels [79]. Tumor angiogenesis has been observed in human melanoma based on experimental [80, 81] and clinical grounds [82]. However, the prognostic value of tumor angiogenesis in melanoma has remained controversial [83]. Several studies have found that increased tumor microvessel density has been correlated decreased disease-free and overall survival [84, 85]. On the other hand, several reports showed no significant differences between tumor microvessel density in metastasizing and nonmetastasizing melanomas [86]. Thus, the potential prognostic value of tumor vascularization in melanoma is unclear at this time [45]. On the other hand, cancer lymphangiogenesis based on experimental and clinical data has been found to be significantly correlated with poor prognosis as presented in this review article. Such a strong evidence indicates that the most likely mechanism of initial spread is through the lymphatic system. In view of the well-established phenomenon of lymhangiogenesis and the strong clinical data of SLN from melanoma and breast cancer as a significant prognostic biomarker, the lymphatic system should be considered a major gateway for cancer to spread.

## Comparative genetics of lymph node and distant metastases

### Kamila Naxerova

Metastasis of cancer cells from a primary tumor to a secondary location is always an undesired event, but some forms of metastasis are less ominous than others. Dissemination to and outgrowth in locoregional tumor-draining lymph nodes is still considered a curable disease stage (III), while metastasis to distant organs defines stage IV cancer and is generally approached with palliative treatment only. It is still largely unknown whether the significantly poorer prognosis that is associated with distant metastasis – for example, 5-year survival in stage III colorectal cancer patients is above 50% but drops to 12% in stage IV patients [87] – reflects the unique biology of distant disease, or whether the discrepancy is simply caused by the fact that lymph nodes are not vital organs. It is clear, however, that lymph node and distant metastases form through distinct processes that pose different challenges for cancer cells.

The first difference is that tumor cells generally travel shorter distances to reach a tumor-draining lymph node than to reach a distant site; this may affect their survival probabilities. Secondly, the mechanical stress that migrating tumor cells are exposed to is considerably lower in the lymphatics than in the blood [88]. Thirdly, the chemical composition of the lymph differs from the blood and may directly support survival by protecting tumor cells from oxidative stress, for example [89]. It is likely that these differences cumulatively result in different selection pressures and thereby diverging genetic characteristics of lymph node and distant metastases. For example, distant metastasis may select for particularly resistant clones with superior ability to evade apoptosis in the face of adverse conditions, while a wider range of clones may succeed during the process of lymphatic metastasis.

**Fig. 8:**
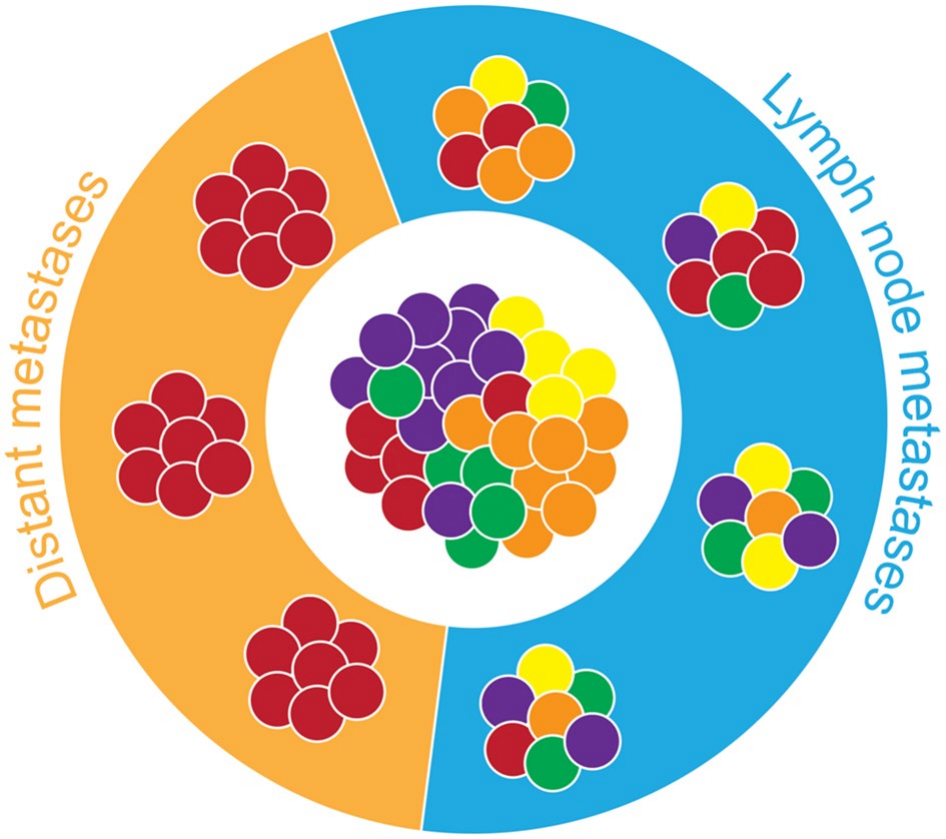
Lymph node metastases mirror the diversity of the primary tumor and are genetically distinct from each other. In contrast, distant metastases are monoclonal and resemble each other

Indeed, recent evidence has confirmed that the genetic composition of individual lymph node metastases is more diverse than the genetic composition of individual distant metastases [90, 91], corroborating the idea that more primary tumor clones can reach or thrive in the lymph node environment than at distant sites. The polyclonality of lymph node metastases [90–92] thus stands in contrast to the more severe heterogeneity reduction that is typically observed in distant metastases [93, 94]. In addition to being polyclonal, lymph node metastases are also polyphyletic with respect to the primary tumor [90]. That is, anatomically distinct lymph node metastases do not necessarily resemble each other—their closest genetic neighbors are not other lymph node metastases but cell populations from various primary tumor regions (Fig. 8). This suggests that i) lymph node metastases often arise independently from each other by separate seeding events originating in the primary tumor and that ii) lymph node metastasis does not select for a particular clone with superior metastatic ability, but many subclones can successfully colonize lymph nodes. For distant liver metastases, the situation is quite different. Not only do they tend to be monoclonal, they are also monophyletic and closely resemble each other [90]. This indicates either successive seeding from metastasis-to-metastasis or strong selective pressures that favor the dissemination and outgrowth of one particular subclone. The trend towards monophyly in distant metastases has not only been shown for liver lesions but appears to apply more broadly [101].

Together, these emerging data suggest that lymph node and distant metastases develop according to different evolutionary rules. This is particularly interesting to consider in light of the traditional view that lymph node metastases are instrumental in giving rise to distant metastases. Fueled by the observation that patients with lymph node metastasis have a worse prognosis than patients with tumor-free lymph nodes, a classic hypothesis states that lymph node metastases represent an important intermediary stage of systemic disease development and that they are causally implicated in seeding distant metastases [95]. If that were true, the two metastasis types should be connected by a common evolutionary origin. Again, comparative genetics can provide some answers in this respect. The consensus of multiple human studies is that overall, there is no evidence of a particularly close genetic connection between lymphatic and distant metastases. Independent investigations have consistently found that lymph node and distant metastases share a common evolutionary origin in approximately 25–35% of cases, and are independently derived from the primary tumor in the remaining 65–75% [96–99].

With the important caveat that metastasis seeder cells may still have traveled through the lymphatic system on their way to distant sites [100], or that specific distant metastasis-seeding subclones reside in the lymph node at low frequencies that evade detection, these data indicate that lymph node and distant metastases are rather distinct entities from a genetic perspective. They are formed according to different evolutionary rules and do not appear to share a common origin in most cases. Larger-scale investigation of human metastasis samples across different tumor types and clinical scenarios will undoubtedly further increase our understanding of the differences between these metastasis types in the future.

## Cancer micrometastasis and dormancy

### Laura Keller and Klaus Pantel

The metastatic cascade is a general denomination of the process in which tumor cells leave the primary tumor, then intravasate into the blood vessels, travel as circulating tumor cells (CTCs) through the bloodstream and finally reach secondary organs where they first settle as disseminated tumor cells (DTCs) and eventually grow into micro-metastases and/or overt macro-metastasis. Here, we will present how the study of CTCs and DTCs that represent two distinct steps of the metastatic cascade can contribute to a better understanding of cancer dissemination and metastasis formation.

### Circulating tumor cells (CTCs)

CTCs are tumor cells derived from a tumor lesion (primary and/or metastases) present in the peripheral blood. CTCs can be captured by a simple blood draw and this non-invasive, easy way to get “real” tumor material is pivotal to be able to better understand cancer progression, which can contribute to improve the survival of patients with solid malignancies. CTC detection therefore constitutes the core of the so-called liquid biopsy concept introduced 10 years ago [101], and the clinical applications of CTC detection and characterization have over the past years tremendously increased. CTC detection contributes to personalized medicine in oncology by providing a better definition of the prognosis of the patients, by enabling the characterization of resistance mechanisms to therapy and tumor heterogeneity, the early detection of progression in the metastatic setting and the detection of minimal residual disease in the earlier stages.

In the understanding of the complexity of metastatic spread directions, CTC detected in mouse models of cancer dissemination are helpful tools. In patients with affected lymph nodes, CTC detection launches new interesting avenues for minimally invasive detection of a sub-clinical tumor cell dissemination [105, 106], and their genetic characterization could also provide missing pieces in the understanding of lymph node role in distant metastasis seeding [96]. Interestingly, genomic characterization of CTCs has already enabled the follow up of tumor clonal evolution during the metastatic cascade in colorectal cancer patients [107], which brings new elements in the development of intra-tumor heterogeneity. In a mouse model of melanoma dissemination, the metabolic characterization of CTCs has recently contributed to discover how lymphatic dissemination can “prime” CTCs to survive to the oxidative stress in the blood circulation [89]. Brain tumors and brain metastasis present one of the deadliest outcomes. Interestingly, CTCs can be detected in the peripheral blood of patients with brain metastasis and with glioblastoma, providing a precious access to tumor material for clinical management [108, 109]. From a biological point of view, these results show that the blood brain barrier is therefore permeable to cell dissemination, and a recent study in patients with medulloblastoma has unexpectedly suggested that hematogenous dissemination might be even the privileged route for loco-regional dissemination in these patients [110].

**Fig. 9:**
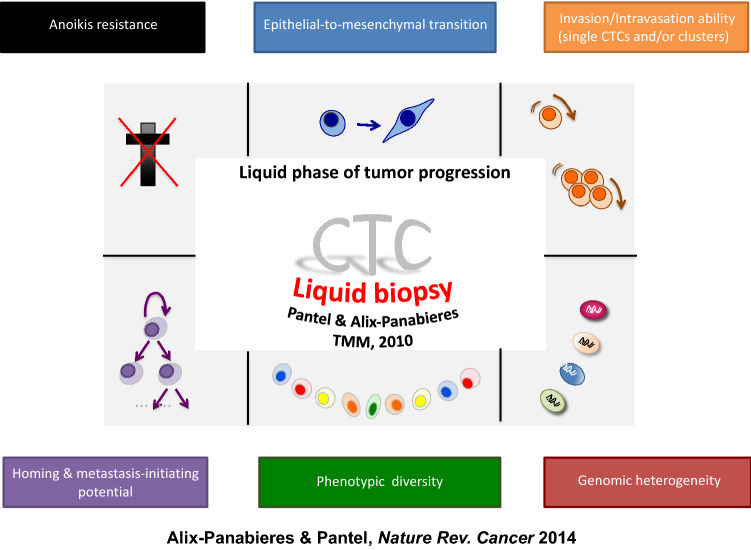
CTC Biological aspects, adapted from Figure 3 of a previous publication, Alix-Panabieres and Pantel, Nature Review Cancer 2014

With the ability to study CTC biology at the single cell level, new insights in the hematogenous dissemination process are also regularly unraveled (Fig. 9). CTCs have to adapt in blood to harsh conditions to be able to survive and finally metastasize. They have to develop strategies to fight against different types of death (anoikis induced by a lack of interaction with extra-cellular matrix or ferroptosis induced by oxidative stress). Single cell mode of dissemination has been extensively studied, notably with the acquisition of a mesenchymal, motile phenotype through epithelial mesenchymal transition (EMT). However, the clinical relevance of mesenchymal CTCs is still not completely clear in comparison to the large amount of prognostic data generated in patients with epithelial CTCs [111, 112]. There is also a lack of comprehensive genomic characterization of “mesenchymal CTCs” that are often phenotypically characterized with non-specific markers like Vimentin that are also shared by leukocytes. The dissemination of single cell phenotypic and genotypic characterization methods will enable to launch larger studies on patients in the future aimed to decipher important questions related to EMT, such as, which degree of mesenchymal de-differentiation is necessary for the cells to survive in the blood flow, which kind of benefit have the cells to acquire this de-differentiated phenotype like escape from the surveillance of immune cells or the development of metastasis initiating capabilities. Next to single cell mode of dissemination, it seems that collective dissemination via clusters of CTCs could also provide substantial survival advantages. Interestingly, the clusters reported in the peripheral blood of cancer patients are not systematically homogenous and can be also composed of leukocytes [113, 114]. The interaction between leukocytes and CTCs can induce an epigenetic reprogramming of CTC that provides survival benefit. These insights could provide interesting new therapeutic strategies based on disruption of metastasis capacity.

CTC derived models (CTC-derived cell lines or CTC derived xenografts) are rare, difficult to establish (they are often derived from exceptional patients with very high number of CTCs) but are especially interesting for launching functional assays to study the biology of CTCs or test in vitro and in vivo pharmacological drugs [115, 116].

CTC detection should also be considered to evaluate the safety of invasive diagnosis procedures like tumor biopsies or surgery [117]. Indeed, it is currently unknown whether the mobilization of CTC during these procedures can lead to an important blood-born dissemination and subsequent outgrowth of tumor cells, responsible of relapse. Built on this hypothesis, a clinical trial has been successfully granted by an ERC (INJURMET) to decipher this important, provocative question.

### Cancer dormancy and disseminated tumor cells (DTCs)

**Fig. 10:**
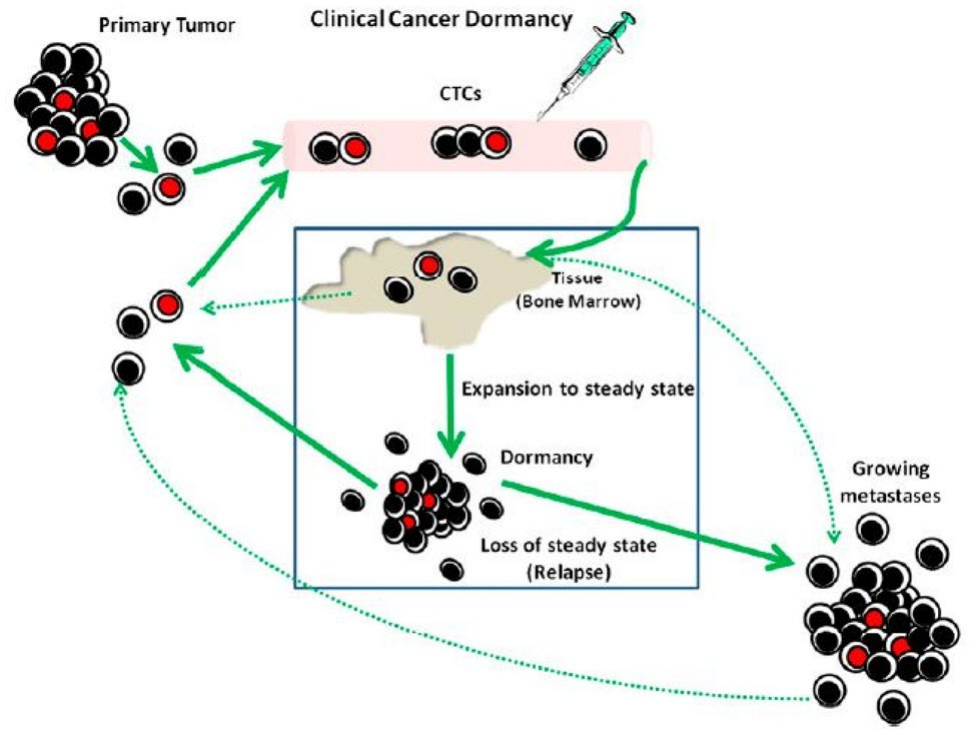
Diagram of clinical cancer dormancy. Cells from the primary tumor are shed into the circulation (CTCs) and a small percentage lodge in tissue including bone marrow. A portion of the CTCs are in cell-cycle arrest and are chemo- and radio-resistant, that is, stem-like cells. These are colored red. Later, these initial cells, a differentiated subset of them, or a different cell type proliferate and establish micrometastases in tissue. There is a steady-state balance between cell proliferation and cell death. Some of the cells that are destined to die are shed into the blood (CTCs). In some patients, the balance changes and metastatic growth occurs (relapse). Solid lines represent dormancy pathways; dotted lines represent pathways for conventional metastatic growth [130]. Permission has been obtained from Proc Natl Acad Sci to reproduce this figure as Fig. 10 [130]

The definition of clinical cancer dormancy is not well established. In general, it is agreed that there is an unusually long time between resection of the primary cancer and subsequent relapse of the disease for a patient who has been clinically free of disease by physical examination, imaging and laboratory studies. Figure 10 shows the current concept of cancer dormancy. This condition is observed in certain carcinomas (e.g., estrogen receptor-positive breast cancer) with relapse occurring 5–25 years later [118]. Cancer dormancy is certainly different from a “cure” in which all cancer cells have been eliminated. However, the difficulty is that cancer dormancy and “cure’ can only be distinguished retrospectively, as to date no clinical criteria nor biological biomarkers have been established to define cancer dormancy. Thus, there is a major effort to characterize the molecular mechanisms responsible for maintaining tumor cell dormancy and identify biomarkers indicating the presence of dormant cancer cells in patients.

DTCs are subject of choice for cancer dormancy study [119]. Once CTCs have successfully extravasated, they settle in distant organs as disseminated tumor cells forming micro-metastases. Bone marrow aspirates can be easily analyzed for DTC presence by immunocytochemical detection of epithelial keratins [112]. In bone marrow, DTCs can be found in a substantial proportion of patients clinically considered as metastatic free [120] rendering bone marrow as a putative reservoir for DTCs. Interestingly, DTC detection in bone marrow correlates with metastatic and loco-regional relapses. However, a substantial fraction of patients with DTCs at initial surgery show no relapse after 10 years of follow up [120], which is consistent with the dormancy concept. Indeed, DTC are mostly non-proliferative (Ki67 negative), have a cancer stem cell phenotype (CD44+/CD24−) and present a down regulation of MHC class of antigen on their surface [121]. Still many questions are associated to the dormancy concept, about the host factors (genetic or not) that can induce or break dormancy, about the interplay with the immune system, about the molecular mechanisms that are involved in the break of dormancy, or about the effects of current therapies on dormant cells [122]. Hormone receptor-positive breast cancer is particularly concerned by relapses after 10 years of treatment [118]. In the attempt to identify DTC-associated genes, we compared the expression profile of early breast tumors presenting DTCs in bone marrow to breast tumors without DTCs. Among the differently expressed genes, we identified *RAI-2* as a potential new metastasis-suppressor gene [123]; loss of RAI2 induces a transcriptional reprogramming leading to a higher expression of genes implicated in mesenchymal phenotype like vimentin, ZEB1, SNAI1, SNAI2. In parallel, we also have established DTC-derived cell lines, and showed how DTC with a stemness phenotype can adapt to hypoxic conditions in bone marrow by the induction of the unfolded protein response [124]. This functional model will also be crucial to better understand the biology of DTCs.

### Concluding remarks

The liquid biopsy field requires joint efforts from both academic and industrial institutions to make promising cancer biomarkers like DTCs, CTCs, cell-free nucleic acids, extracellular vesicles or tumor-educated platelets fit for use in clinical practice to improve the survival and quality of life for cancer patients [104, 125]. Liquid biopsy may be an important tool to define and follow cancer dormancy. To fulfill this aim, European consortia have been established, first CANCER-ID (2015–2019) and now the European Liquid Biopsy [122]Society (start in 2019, www.elbs.eu). Presently ELBS gathers 50 institutions and is a founding member of the International Liquid Biopsy Standardization Alliance (ILSA) coordinated by the Foundation of the National Institute of Health, USA [126]. CTC and DTC monitoring of cancer patients may further characterize the status of cancer dormancy [103, 122].

## Summary and future perspectives

### Marlys Witte

Historically, for centuries preceding our current concepts of cancer spread, a fundamental link to the lymphatic system was recognized, first in the “lymph theory” of the origin of cancer from tissue fluid (now revisited in the cancer “microenvironment”) and subsequently by Virchow of the cell theory [127]. In addition to formulating the cellular theory of cancer, he also described the barrier function of lymph nodes but, in addition, their deleterious role as a nidus for proliferation and subsequent spread of cancer cells and their “poisonous products” [128]. Surgeons with radiotherapists took the next major steps in confronting cancer metastasis first with radical extirpation of the primary tumor and extensive lymphadenectomy with or without radiotherapy, later modified, and subsequently tailored to the sentinel lymph node approach to assess and, if appropriate, to take further steps to abort lymphogenous spread. Adjuvant chemotherapy and more recently immunotherapy were added as concepts of cancer metastasis evolved.

As described in this Conference session and other related sessions, fundamental questions about cancer metastasis remain unanswered, whether via a lymphogenous or hematogenous route, the underlying mechanisms and pathways of cancer cell entry into either vasculature, functional, alterations in the extracellular matrix including physical forces promoting cell growth and migration, vascular mimicry by tumors, molecular events during cancer cell transit, the fine line between benign metastasizing tumors and true malignancies, the basis for cancer latency, specific sites of spread, and resistance or susceptibility to different forms of treatment.

A major focus of current research has been the role of angiogenesis (both of blood vessels and of lymphatics) and angiogenic products in promoting tumor growth and as a therapeutic target. Indeed, until relatively recently, the very existence of tumor lymphangiogenesis was questioned [129]. However, mounting evidence summarized earlier has elucidated a crucial role for tumor lymphangiogenesis and its molecular basis (specifically under the control of the VEGF, particularly VEGF-C, and the angiopoietin-tie family of context-dependent growth factor ligands and receptors) within and around the primary tumor (intratumoral and peritumoral lymphatics), as well as in draining lymph node, and distant sites (pre-metastatic niches) even before tumor cells have arrived. Interactions with distinct immune cell populations at each such site are under study and translation into the clinic as biomarkers and immunotherapy.

The process of epithelial/endothelial-mesenchymal transition in promoting migration of stable stationary cell types along with its molecular mediators is being dissected not only in relation to cancer spread but also in embryonic development. Specifically, genes, proteins (e.g. transcription factors, gap junction proteins, mechanotransducers) and VEGF and angiopoietin signaling pathways have been implicated in development encompassing vasculogenesis and angiogenesis. Molecules and mechanisms identified in abnormal development of the lymphatic vasculature and associated lymphoid organs in human lymphatic disorders (e.g. hereditary lymphedema) as well as in mouse models mimicking these conditions have shed unexpected light not only on developmental processes but also found a parallel in cancer spread.

Liquid biopsy sampling peripheral blood is providing a source of circulating cancer cells for detailed study. But liquid biopsy of central thoracic duct lymph could be more revealing of the circulating cancer cells for genomic and other studies as well as for the associated trafficking immune cells and chemical mediators in this central lymph draining the primary tumor and regional lymph nodes of most solid tumors. Thoracic duct lymph can now be accessed minimally invasively through transvalvular endovascular cannulation from a peripheral or central venous route and large volumes collected for study. This lymph is likely a major source of cancer cells circulating in blood in patients with advanced malignancies, mistakenly thought to arise from direct hematogenous spread in the primary tumor or regional lymph nodes.

In summary, clearly major advances have taken place in understanding, development of tools for study including an array of imaging techniques, as well as in targeted therapy (including endolymphatic) of cancer metastasis whether occurring by the lymphogenous or hematogenous route. However, translation of these advances to improved patient outcomes remains the continuing challenge both to basic scientists as well as clinicians.

## Abbreviations

SLN: Sentinel lymph node
VEGF: Vascular endothelial growth factor
LEC: Lymphatic endothelial cell
LYVE-1: Lymphatic vessel endothelial hyaluronan receptor 1
Prox-1: Prospero homeobox protein 1
CCR7 C–C: Chemokine receptor 7
LDL: Low-density lipoprotein
TGF: Transforming growth factor
CTC: Circulating tumor cell
DTC: Disseminated tumor cell
EMT: Epithelial mesenchymal transition
PDL-1: Programmed death-ligand 1
IFN-g: Gamma Interferon-g
